# Survival analysis and influence of the surgical aggression of a cohort of orthopedic and trauma patients in a non-controlled spread COVID-19 scenario

**DOI:** 10.1186/s12891-021-04303-8

**Published:** 2021-06-28

**Authors:** Fernando Corella, Roberto S. Rosales, David Guzman Domenech, Miguel Cañones Martín, Ricardo Larrainzar-Garijo

**Affiliations:** 1 Orthopedic and Trauma Department, Hospital Universitario Infanta Leonor, C/ Gran Vía del Este N° 80, 28031 Madrid, Spain; 2 Surgery Department, School of Medicine, Universidad Complutense de Madrid, Plaza de Ramón y Cajal, s/n, 28040 Madrid, Spain; 3 Unit for Hand & Microsurgery, GECOT, C/ María del Cristo Osuna, 20, 38204 San Cristobal de La Laguna, Santa Cruz de Tenerife, Spain

**Keywords:** Coronavirus, COVID-19, Elective orthopedic surgery, Trauma surgery

## Abstract

**Background:**

Determining the infection rate and mortality probability in healthy patients who have undergone orthopedic and trauma surgeries (OTS) during a period of uncontrolled COVID-19 transmission may help to inform preparations for future waves. This study performed a survival analysis in a cohort of non-infected OTS patients and determined the effect of COVID-19 on mortality.

**Methods:**

This observational study included 184 patients who underwent OTS in the month before surgical activities ceased and before the implementation of special measures. Four groups of surgery (GS) were established based on the location of the surgery and the grade of inflammation produced. Crude risk of infection and infection rates were assessed. Survival and failure functions by GS were analyzed. Comparison of the Kaplan-Meier survival curves by GS was assessed. Cox regression and Fine-Gray models were used to determine the effect of different confounders on mortality.

**Results:**

The crude risk of COVID-19 diagnosis was 14.13% (95% CI: 9.83–19.90%). The total incidence rate was 2.67 (1000 person-days, 95% CI: 1.74–3.91). At the end of follow-up, there was a 94.42% chance of surviving 76 days or more after OTS. The differences in K-M survivor curves by GS indicated that GS 4 presented a lower survival function (Mantel-Cox test, *p* = 0.024; Wilcoxon-Breslow test, *p* = 0.044; Tarone-Ware test, *p* = 0.032). One of the best models to determine the association with mortality was the age-adjusted model for GS, high blood pressure, and respiratory history, with a hazard ratio of 1.112 in Cox regression analysis (95% CI: 1.005–1.230) and a sub hazard ratio of 1.111 (95% CI: 1.046–1.177) in Fine-Gray regression analysis for competitive risk.

**Conclusions:**

The infection risk after OTS was similar to that of the general population in a community transmission area; the grade of surgical aggression did not influence this rate. The survival probability was extremely high if patients had not previously been infected. With higher grades of surgical aggression, the risk of mortality was higher in OTS patients. Adjusting for age and other confounders (e.g., GS, high blood pressure and respiratory history) was associated with higher mortality rates.

**Supplementary Information:**

The online version contains supplementary material available at 10.1186/s12891-021-04303-8.

## Background

On March 11, 2020, the World Health Organization (WHO) declared the novel coronavirus disease 2019 (COVID-19) as a global pandemic. By May 2020, COVID-19 had spread globally [1]. During the first and second (ongoing at the time of writing) waves, Spain became one of the countries with the highest number of cases and Madrid, one of the most affected regions [2].

During the first and second infection waves, elective and urgent orthopedic and trauma surgery (OTS) ceased, as many hospitals were operating with grade 3 COVID-19 occupancy scores [3]. However, normal orthopedic healthcare should be restored between infection waves and in the future. It is therefore essential to determine the risk of virus transmission and mortality following elective and urgent orthopedic and trauma surgery (OTS) in non-infected patients in a community with high COVID-19 transmission levels. Furthermore, it is important to determine whether surgical aggression grade effects these risks.

There are few reports in the literature that examine the risks of infection after performing elective and urgent OTS in a community with high levels of COVID-19 transmission. While several studies have analyzed mortality in this context, reporting high rates [3–5], the majority of patients in these studies had already been infected or were in the incubation period when the surgery was performed.

This study aimed to analyze the risk of infection and perform a survival analysis in a cohort of healthy patients who underwent OTS during a period of uncontrolled COVID-19 transmission – at the start of the first wave, before any control measures had been implemented. Infection and survival rates in OTS patients at this time can be compared to those during periods in which measures have been implemented. The results of this study could be used to guide decision-making between infection waves when elective or trauma surgeries need to be introduced again and to determine which patient and type of surgery has the lowest and highest risk.

## Methods

### Study design

This is an observational study with an ambispective cohort design [6]. It adhered to the Strengthening the Reporting of Observational Studies in Epidemiology (STROBE) checklist [7]. Patients who underwent OTS between February 13 and March 13, 2020 were included in this study. Once the study population was defined in a retrospective manner, the cohort was followed prospectively until May 5, 2020.

### Setting

This study was carried out at the Hospital Universitario Infanta Leonor, located in the south-east side of Madrid City, Spain and covering a target population of 305262 patients. The hospital was at grade 3 COVID-19 occupancy at the time of the study [3]. On March 13, 2020, the occupancy rate reached 202%, and the hospital was declared a “100% COVID-19 hospital” – no other medical or surgical activity apart from the treatment of COVID-19 patients could be carried out.

#### Eligibility criteria

We included all patients who had undergone OTS in the month before surgical activities ceased. During this period, the state of “alert” had not yet been declared; thus, COVID-19-specific infection prevention and control measures had not been established and the rate of transmission was high. Surgical activity was therefore completely normal during this time, mimicking periods between waves or when a new one is about to begin. Patients were recruited by MC and DG via phone call. Prior to the phone interview, patients were asked for their consent to participate in the study.

#### Data collection

General patient data were obtained from the hospital database. Disease histories were obtained from the preoperative evaluation performed by the anesthesiology department. Only patients who were admitted to the hospital had their outcome data recorded on the electronic medical record. For the patients who were not admitted, outcomes were confirmed through a structured phone call interview, performed within 2 weeks for all patients to avoid any discrepancy in exposure (Additional file 1).

#### Ethics

The study strictly followed the ethical principles of biomedical research. The use of clinical/personal patient data in the research was approved by the Ethics Committee of the hospital (R-026-20).

### Participants

The inclusion criteria were pre-anesthetic assessment approval (no suspicion of COVID-19 or COVID-19 exposure during the preoperative anesthetic evaluation) and an age over 18 years. The exclusion criteria were the presence of symptoms compatible with COVID-19 and the absence of consent to participate in the study.

### Variables and data sources

The primary outcomes of the study were diagnosis of COVID-19 and death from COVID-19. Case definitions were classified as recommended by the Spanish Ministry of Health [8] and the WHO [9]. The independent and potential confounders were: “Age”, “Sex”, “Body Mass Index” (BMI), “High Blood Pressure” (HBP), “Diabetes” (DM), “History of Respiratory Disease” (RespHist), “History of Cardiological Disease” (CardiolHist), “History of Kidney Disease” (RenalHist), “American Society of Anesthesiologists Physical Status Classification System” (ASA), and “Group of Surgery” (GS).

The “Group of Surgery” variable was based on the grade of aggression and inflammation. Four different groups were established based on the location of the surgery and the grade of the surgery taking the NICE guidelines into account [10]. Groups 1 and 2 included procedures performed in small joints (foot, ankle, hand and wrist) while Groups 3 and 4 included those performed on big joints (elbow, shoulder, knee and hip). The difference between Groups 1–2 and 3–4 was the grade of aggression of the procedure (1 and 3: soft tissue and arthroscopic procedures; 2 and 4 bone and arthroplasty procedures; Table 1).

**Table 1:** Groups of Surgery based on the location of the surgery and aggression and inflammation grade

Groups of Surgery	
**Group 1:** soft tissue and arthroscopic procedures in small joints (foot, ankle, hand and wrist)	
**Group 2:** bone and arthroplasty procedures in small joints	
**Group 3:** soft tissue and arthroscopic procedures in big joints (elbow, shoulder, knee and hip)	
**Group 4:** bone and arthroplasty procedures in big joints	

### Statistical analysis

#### Crude rates

The crude risk and risk ratio (RR) of COVID-19 diagnosis were determined by case definition and GS, and the corresponding 95% confidence intervals (CI) were calculated. The crude incidence rate (person-day) and crude incidence rate ratio (IRR) of COVID-19 diagnosis by GS and their 95% CI were obtained. RR and IRR were analyzed using GS 1 as a reference.

#### Survival and hazard function analysis

The survival function of the entire cohort was analyzed by Kaplan-Meier (K-M) [11] and life-table actuarial methods. Survival and failure functions by GS were analyzed by K-M and competing risk models [12]. Comparison of the K-M survival curves by GS was assessed using the Mantel-Cox log rank test (M-C) [13], the Wilcoxon-Breslow test [14], and the Tarone-Ware test (T-W) [15], which followed the chi-squared distribution under the null hypothesis (Ho) that the survival functions are equal and a level of significance (α) of 0.05.

#### Quality of follow-up

The distribution of the frequencies of the different states (Alive, Dead, Lost) at the end of the follow-up was calculated. The follow-up period for the different states at the last observation in the cohort was analyzed as another indicator of the quality of the follow-up. Finally, the minimum, maximum, mean, median, 25th, and 75th percentiles were assessed by the different states at the end of the follow-up.

#### Multivariate survival analysis for measuring the effect

##### Selection of independent variables

All independent variables (IVs) were assessed by univariate Cox regression analysis and those with *p* ≤ 0.20 were considered to be confounders [16]. BMI and ASA were excluded based on the univariate analysis (complete data in Additional file 2). “RespHist” (*p* = 0.989) with a non-significant hazard ratio (HR) was included in the multivariate analysis because of its theoretical importance. The final IVs selected were as follows: “Age,” “GS,” “HBP,” “DM,” “RespHist,” “CardiolHist,” and “RenalHist.”

##### Maximum model (MMax)

Based on previous publications [17], the variable “Age” was selected as the main predictor variable and the rest of the IVs were considered to be possible confounders. The final reference model (MMax) was built up based on the confounders and their interaction with the predictor variable.

##### Assessment of the interactions

The interactions were assessed using a chunk test, which compared the reference model (MMax) to the model without the interactions (MMaxNoInteract). It was based on the likelihood ratio statistic that followed the chi-squared distribution with the degrees of freedom equal to k (number of IVs) minus the number of interactions, and a level of significance of 0.05 (α = 0.05; complete data in Additional file 2).

##### Assessment of the confounders

In line with the Maldonado and Greenland recommendations [16], confounders were assessed based on the change in their effect or HR being less than 10% compared with the reference model. The final models were compared using the Stata user command “confound” [18] developed for modeling confounding in linear, logistic, and Cox regression (complete data in Additional file 2).

##### Final model assessment

The final selected models adjusted by confounders were analyzed using a Cox regression model and a Fine-Gray Regression model [19–21] for competing risks, which showed the effect based on the sub hazard ratio (SHR).

##### Diagnosis of the model

Two assumptions are required in the Cox proportional hazard model: the proportional assumption and the log-linear relationship. The proportionality assumption assumes that the effect of the predictors on the hazard rate is constant throughout the follow-up time. To verify this assumption, the interaction of each predictor with the survival time was added to the Cox model chosen to measure the effect. The null hypothesis was established, which assumed that the coefficients of interaction terms were statistically equal to zero. Finally, the relationship between the Schoenfeld residuals and survival time was analyzed using a chi-squared test, assuming proportionality when *p* > 0.05. The log-linear relationship assumption of the Cox model assumes that the relationship between the instantaneous IR and the explanatory variables is log-linear. The analysis of the squared linear predictor was used in this way to confirm that the squared predictor coefficient was not significant (p > 0.05).

## Results

Only one patient was excluded from this study because verbal consent was not obtained. In total, 184 patients were recruited for this study, none of whom dropped out at follow-up. Demographic data and the distribution of the IVs are shown in Table 2.

**Table 2:** Demographic data and independent variables distribution

	Entire Cohort		Small Joints (soft tiss./arthros.)		Small Joints (bone or arthrop.)		Big Joints (soft tiss./arthros.)		Big Joints (bone or arthrop.)	
**Age,** ***N*, mean (SD)**	*184*	60.04 (18.81)	*44*	53.39 (16.34)	*54*	52.80 (15.21)	*21*	52.72 (15.18)	*65*	72.92 (17.57)
**Sex male,** ***n/N***	*81/184*	44.02%	*22/44*	50.00%	*18/54*	33.33%	*13/21*	61.90%	*28/65*	43.08%
**High blood pressure,** ***n/N***	*74/184*	40.22%	*13/44*	29.55%	*16/54*	29.63%	*5/21*	23.81%	*40/65*	61.54%
**DM,** ***n/N***	*24/184*	13.04%	*6/44*	13.64%	*1/54*	1.85%	*3/21*	14.29%	*14/65*	21.53%
**History of Renal disease,** ***n/N***	*11/184*	5.98%	*0/44*	0.00%	*0/54*	0.00%	*0/21*	0.00%	*11/65*	16.92%
**History of Cardiological disease,** ***n/N***	*26/184*	14.13%	*2/44*	4.55%	*3/54*	5.56%	*1/21*	4.76%	*20/65*	30.77%
**History of Respiratory disease,** ***n/N***	*38/184*	20.65%	*5/44*	11.36%	*14/54*	25.93%	*3/21*	14.29%	*16/65*	24.62%
**BMI,** ***N,*** **mean (SD)**	*175*	28.53 (5.64)	*42*	28.52 (5.84)	*52*	28.42 (6.26)	*20*	29.38 (4.65)	*61*	28.34 (5.33)
**ASA Classification** ***N***										
**I**	*27/184*	14.67%	*9*	33.33%	*9*	33.33%	*3*	11.11%	*6*	22.22%
**II**	*119/184*	64.67%	*31*	26.05%	*42*	35.29%	*16*	13.45%	*30*	25.21%
**III**	*32/184*	17.39%	*3*	9.38%	*3*	9.38%	*2*	6.25%	*24*	75%
**IV**	*6/184*	3.26%	*1*	16.67%	*0*	0.00%	*0*	0.00%	*5*	83.33%

The total number of missing values was 0.41% (9/184 missing BMI values); thus, it is unlikely that missing values affected the results [22].

The quality of the follow-up was classified as excellent. No patients had dropped out of the study by the end of the last observation. The mean follow-up time was 32.8 days.

The overall crude risk of COVID-19 diagnosis according to the four different GS is shown in Table 3. The relative risk of COVID-19 diagnosis by GS, taking GS 1 as reference, were not significant independently, both including or excluding the suspected cases (complete data in Additional file 3).

**Table 3:** Risks of COVID-19 diagnosis

	Cases	***N***	Risk	Wilson (95% Conf. Interval)		RR	(95% CI)	
**a) Risk of COVID diagnosis by Group of Surgery**								
**Small Joints (soft tiss./arthros.)**	8	44	18.18%	9.51%	31.96%	1	NA	NA
**Small Joints (bone or arthrop.)**	7	54	12.96%	6.42%	24.42%	0.71	0.28	1.81
**Big Joints (soft tiss./arthros.)**	0	21	0.00%	0.00%	15.46%	0.12^a^	0.01	1.99
**Big Joints (bone or arthrop.)**	11	65	16.92%	9.72%	27.82%	0.93	0.41	2.13
**Total**	26	184	14.13%	9.83%	19.90%			
**b) Risk of COVID diagnosis (only probable and confirmed cases) by Group of Surgery**								
**Small Joints (soft tiss./arthros.)**	1	44	2.27%	0.40%	11.81%	1	NA	NA
**Small Joints (bone or arthrop.)**	2	54	3.70%	1.02%	12.54%	1.63	0.15	17.38
**Big Joints (soft tiss./arthros.)**	0	21	0.00%	0.00%	15.46%	0.68^a^	0.03	16.07
**Big Joints (bone or arthrop.)**	9	65	13.85%	7.46%	24.27%	6.09	0.80	46.40
**Total**	12	184	6.52%	3.77%	11.05%			

^a^In case of zero event, the RR was computed with a constant continuity correction (k = 0.5)

*RRs* Rrelative risks

The total IR and the crude IR (1000 person-days) of COVID-19 diagnosis by GS are shown in Table 4. There were no statistical differences in the IRR when using GS 1 as reference.

**Table 4:** Incidence rate and incidence rate ratio of COVID-19 diagnosis

IR and IRR of COVID diagnosis by Group of Surgery								
	person-time	***Failures***	IR (1000 person-day)	Poisson-Exact (95% Conf. Interval)		IRR	Exact (95% CI)	
**Small Joints (soft tiss./arthros.)**	2254	8	3.55	1.53	6.99	1	.	.
**Small Joints (bone or arthrop.)**	2984	7	2.35	0.94	4.83	0.66	0.24	1.82
**Big Joints (soft tiss./arthros.)**	1031	0	0	0	3.58^a^	0		
**Big Joints (bone or arthrop.)**	3469	11	3.17	1.58	5.67	0.89	0.36	2.22
**Total**	9738	26	2.67	1.74	3.91			

^a^One-sided, 97.5% confidence interval for zero event

*IR* Incidence rate, *IRR* Incidence rate ratio

Of the 26 patients with COVID-19 diagnosis, 16/184 (8.7%) presented with mild symptoms, stayed at home, and did not require any specific drug treatment while 5/184 (2.72%) were admitted to hospital due to more severe symptoms; all patients survived. There were 7/184 (3.80%) deaths in the entire cohort, 5 of them (2.72%) were deaths that fit the criteria of COVID-19 deaths and 2 (1.09%) were deaths not caused by COVID-19 (complete data in Additional file 4).

The cumulative survival probabilities of the entire cohort calculated using the K-M method and actuarial method are shown in Table 5 (complete data in Additional file 5). At the end of the follow-up, there was a 94.42% chance of surviving 76 days or more after OTS. The actuarial method showed that most of the COVID-19 deaths occurred during the first 1.5 months after surgery. The cumulative probability of survival between 60 and 75 days was 0.957.

**Table 5:** Survival function of entire cohort

**a) Survival function. Kaplan-Meier method. Entire Cohort**						
**Time**	***Beg.***	***Deaths***	***Net Lost***	**Survival probability**	**[95% Conf. Int.]**	
2	184	1	0	0.9946	0.9621	0.9992
19	183	1	1	0.9891	0.9572	0.9973
30	180	0	1	0.9891	0.9572	0.9973
31	179	1	0	0.9836	0.9500	0.9947
40	178	0	4	0.9836	0.9500	0.9947
41	174	1	1	0.9780	0.9423	0.9917
44	164	0	15	0.9780	0.9423	0.9917
60	84	0	2	0.9780	0.9423	0.9917
70	37	0	8	0.9780	0.9423	0.9917
71	29	1	11	0.9442	0.8172	0.9838
76	10	0	10	0.9442	0.8172	0.9838
**b) Survival function. Actuarial Method. Entire Cohort**						
**Interval days**	***Beg.***	***Deaths***	***Lost***	**Survival probability**	**[95% Conf. Int.]**	
0 15	184	1	0	0.9946	0.9621	0.9992
15 30	183	1	2	0.9891	0.9571	0.9973
30 45	180	2	29	0.9771	0.9402	0.9914
45 60	149	0	65	0.9771	0.9402	0.9914
60 75	84	1	71	0.9570	0.8805	0.9849
75 90	12	0	12	0.9570	0.8805	0.9849

The failure function (cumulative incidence of mortality) in the entire cohort calculated by using the K-M method and competing risk method are shown in Table 6 (complete data in Additional file 6). Our data suggest that there was approximately 5.5% chance of dying by COVID-19 in the first 71 days after OTS.

**Table 6:** Failure function. Entire cohort

Failure function. Entire cohort											
Kaplan-Meier method							Competing Risk method				
Time	***Beg.***	***Deaths***	***Net Lost***	Failure function	[95% Conf. Int.]		EndStatMort	Time	R (IA1c)	(95% Conf. Interval)	
2	184	1	0	0.0054	0.0008	0.0379	Dead by COVID	2	0.0054	0.0005	0.0277
19	183	1	1	0.0109	0.0027	0.0428	Dead by COVID	19	0.0109	0.0022	0.0357
28	181	0	1	0.0109	0.0027	0.0428	Dead (other cause)	28	0.0109	0.0022	0.0357
31	179	1	0	0.0164	0.0053	0.0500	Dead by COVID	31	0.0163	0.0045	0.0436
41	174	1	1	0.0220	0.0083	0.0577	Dead by COVID	41	0.0219	0.0073	0.0516
71	29	1	11	0.0558	0.0162	0.1828	Dead by COVID	71	0.0553	0.0121	0.1496
76	10	0	10	0.0558	0.0162	0.1828	Alive	76	0.0553	0.0121	0.1496

“EndStaMort”: Status at the last follow-up. “R(IA1c)”: Competing Risk or Cumulative Incidence of mortality by COVID-19 in presence of Competing Events (other casuse of death)

**Fig. 1:**
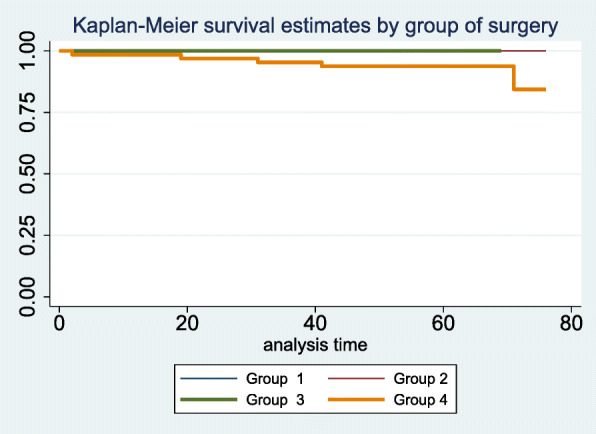
Kaplan-Meier survival by Group of Surgery

**Fig. 2:**
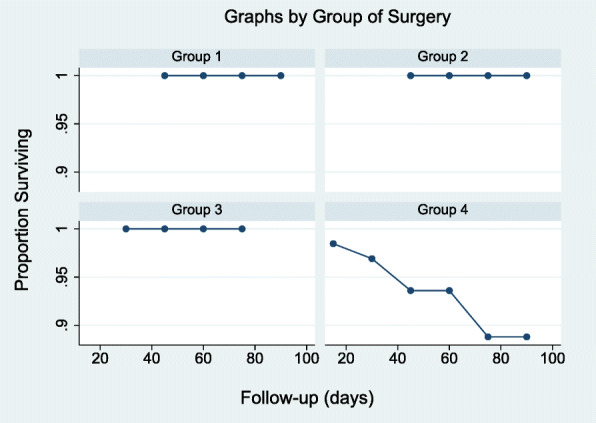
Proportion of survivors by Group of Surgery

The K-M failure function by GS showed that the risk of mortality at 76 days or earlier was 0.1563 (Table 7; complete data in Additional file 6). The comparison of K-M and life tables of survival (actuarial method) curves by GS are shown in Figs. 1 and 2. The differences in survival curves by GS were statistically significant (M-C test, *p* = 0.024; W-B test, *p* = 0.044; T-W test, *p* = 0.032) and determined that GS 4 had a lower survival function compared to the remaining GS (complete data in Additional file 7). One of the five COVID-19 patients who died had undergone arthroplasty for a shoulder fracture, while the remaining four had had hip fractures.

**Table 7:** Failure function by Group of Surgery

Failure function by Group of Surgery						
Kaplan-Meier method						
Time	Beg.	Fail	Lost	Failure function	(95% Conf. Interval)	
**Group 1**						
76	4	0	4	0.00		.
**Group 2**						
76	2	0	2	0.00		.
**Group 3**						
76	1	0	1	0.00		.
**Group 4**						
2	65	1	0	0.0154	0.0022	0.1042
19	64	1	0	0.0308	0.0078	0.1175
28	63	0	1	0.0308	0.0078	0.1175
31	62	1	0	0.0464	0.0152	0.1370
41	59	1	0	0.0626	0.0239	0.1582
71	10	1	3	0.1563	0.0465	0.4545
76	4	0	4	0.1563	0.0465	0.4545

The selected models, adjusted by confounders and analyzed using a Cox regression model (effect based on HR) and Fine-Gray Regression (effect based on SR) model, demonstrated that “Age” adjusted for “HBP” showed the highest association with mortality by COVID-19 (HR: 1.145; SHR: 1.144). The model “Age” adjusted for “GS,” “HBP,” and “RespHist” was also significantly associated with mortality by COVID-19, albeit to a lesser extent (HR and SHR: 1.111; Table 8). The diagnosis of the models demonstrated that the proportional hazard assumption was accepted based on Schoenfeld residuals (*p* = 0.405) and the test of interaction between survival time and the independent variables (*p* = 0.857). The log linear assumption was also accepted (*p* = 0.406; complete data in Additional file 8).

**Table 8:** Final selected models adjusted by confounders

Model	Cox Regression				Fine-Gray Regression			
	HR	***p value***	(95% Conf. Interval)		SHR	***p value***	(95% Conf. Interval)	
**Age Adjusted for: “Group of Surg”, “HBP,”** “**RespHist”**	1.112	0.039	1.005	1.230	1.111	0.000	1.049	1.176
**Age Adjusted for: “Group of Surg”**	1.112	0.042	1.004	1.232	1.11	0.001	1.046	1.184
**Age Adjusted for: “HBP”**	1.145	0.005	1.042	1.257	1.143	0.000	1.088	1.201
**Age Adjusted for: “RespHist”**	1.145	0.003	1.046	1.254	1.144	0.000	1.088	1.202

## Discussion

This study demonstrated that the risk and IR of COVID-19 infection in patients who underwent OTS were similar for the different GSs. However, the cumulative survival probability in patients who had bone and arthroplasty procedures in big joints (GS 4) was significantly lower compared to the rest of the surgery groups; there was a 15.63% chance of dying by COVID-19 by day 76 following surgery. Age adjusted by GS, HBP, and RespHist was associated to the mortality. At the end of the follow-up, there was a 94.42% chance of surviving 76 days or more after OTS.

The risk of COVID-19 diagnosis (considering all types of diagnose) was 14.13% (95% CI: 9.83 to 19.90%) for the entire cohort. In Madrid, which has a population of 6663394 [23], there were 62989 confirmed cases (0.95%) on May 5, 2020; 40851 had been admitted to hospital (64.86% of all cases; 0.61% of the entire population) and 8420 had died (13.37% of all cases; 0.13% of the entire population) [24]. A seroprevalence study performed during the same period in Spain determined that the rate of infection was 11.3% (95% CI: 9.8 to 13.0) and 19.7%, with the consideration of suspected cases. Considering these data, the risk of diagnosis, including suspected cases, in our cohort is similar to the general population.

The relative risk of all COVID-19 diagnoses by the different GSs was not statistically significant when compared using group 1 as the reference. Thus, the grade of aggression and inflammation cannot be considered as a factor of a higher risk of infection in our cohort.

Notably, the most interesting variable to analyze is the probability of survival. In our study, the chance of survival at 76 days or more in a patient who had OTS was 94.42% (95% CI: 81.72–98.38). This high survival probability provides a more positive outlook when compared to results obtained in previous studies. Indeed, Lei et al. [4] reported a mortality rate of 20.5% after elective surgery; the COVID Surg Collaborative group showed a crude 30-day mortality rate of 23.8% in the entire cohort and of 28.8% in the orthopedic surgery cohort. In those studies, all patients had developed the disease by day 5 following surgery, or the COVID-19 infection was confirmed preoperatively thus, they had already been infected. This may be the most important difference; the patients in our study were healthy (not infected) before surgery. The mortality probability after having an OTS, even in an uncontrolled transmission area, is low if the patient has not been infected previously.

Regarding the influence of the aggression and inflammation of the OTS on mortality, there were no deaths in GS 1, 2 and 3. All deaths were concentrated in GS 4 and the lower survival function was statistically significant in this group. Lei et al. [4] concluded that a more complex surgery increases the risk of having the worst evolution; however, they detected several variables (older age and comorbidities) that are more frequent in this group, although they did not include a multivariate model to adjust for confounders. The higher mortality rate in older patients has been reported in several studies with a high number of patients [25–28], mirroring our model in which age was the most important variable. However, most of these studies considered age in addition to the risk factors from unadjusted studies for other confounders [29]. Age adjusted for the GS, HBP, and RespHist was one of the best models for explaining the association of mortality by COVID-19 in patients who underwent OTS, with a confirmed proportional hazard and log-linear assumptions that required the model diagnosis.

This study has some limitations. We could not define a nonsurgical subgroup in our cohort during the same recruitment period and not all types of OTS, such as spine surgery or pediatric surgery, were included as they are performed in another hospital. Representativeness of the general OTS population and changes in case definitions ﻿should be considered when making inferences from the risk and incidence rates of infection by COVID-19 observed in our cohort [30]. This is important because the rates of infection and mortality can be compared in different scenarios and with different infection prevention measures in place. Furthermore, this study is limited by the fact that COVID-19 case definition criteria and testing are still evolving.

The highlight of this observational study is the fact that the patients (who did not have COVID-19) underwent OTS during a period of uncontrolled COVID-19 spread; thus, these data could help clarify the risk of infection and survival probability and aid decision-making between infection waves.

## Conclusions

The infection risk after OTS is similar to that of the general population in a community transmission area; the grade of surgical aggression did not influence this rate. The survival probability is extremely high if patients have not previously been infected with COVID-19. Mortality is higher if the group had a higher aggression and inflammation grade. Age adjusted for confounders such as GS, high blood pressure, and respiratory history is associated with mortality.

## Supplementary Information

**Additional file 1.** Interview guide. Document use by the researches to guide the telephone interview.

**Additional file 2.** Multivariate analysis. The complete STATA data are shown for the following: A Selection of variables. Univariate Cox regression analysis. B Analysis of the confounders and interactions. C Confound assessment.

**Additional file 3.** Relative risks of COVID-19 diagnosis by Group of Surgery. The complete STATA data are shown for the following: A COVID diagnosis (suspected + probable + confirmed cases). B COVID diagnosis (only probable + confirmed cases).

**Additional file 4.** Distribution of cases in the entire cohort. Table showing the distribution of the evolution of cases in the entire cohort (non-infected; alive; COVID-19 deaths; non-COVID-19 deaths; severe disease).

**Additional file 5.** Cumulative survival probabilities of the entire cohort according to the K-M method and the actuarial method. The complete STATA data are shown for the following: A Survival list. Entire cohort. B Actuarial method.

**Additional file 6.** Failure function (cumulative incidence of mortality) in the entire cohort according to the K-M method and the competing risk. The complete STATA data are shown for the following: A Survival function by Group of Surgery. B K-M failure function. Entire cohort. C Failure function. Competing risk. D K-M failure function by Group of Surgery. E Comparison of life tables of survival (actuarial method) by Group of Surgery.

**Additional file 7.** Differences in survival curves by Group of Surgery. The complete STATA data are shown for the following: Log rank, Breslow, and Tare tests for comparing survivor curves by Group of Surgery.

**Additional file 8.** Model assessment. The complete STATA data are shown for the following: A Schonefeld residuals. B Test based on the interaction between survival-time and the independent variables. C Log linear assumption. D Quality of the follow-up.

## Data Availability

The datasets used and/or analyzed during the current study are available from the corresponding author on reasonable request. Most of the materials can be found in the additional material.

## Abbreviations

OTS: Orthopedic and trauma surgeries
STROBE: Strengthening the Reporting of Observational Studies in Epidemiology
BMI: Body Mass Index
HBP: High Blood Pressure
DM: Diabetes Mellitus
RespHist: History of Respiratory Disease
CardiolHist: History of Cardiological Disease
RenalHist: History of Kidney Disease
ASA: American Society of Anesthesiologists Physical Status Classification System
GS: Group of Surgery
NICE: National Institute for Health and Care Excellence
CI: Confidence intervals
IR: Incidence rate
RR: Relative risks
IRR: Incidence rate ratio
K-M: Kaplan-Meier
M-C: Mantel-Cox log rank test
T-W: Tarone-Ware test
IV: Independent variables
HR: Hazard ratio
MMax: Maximum model
MMaxNoInteract: Model without the interactions
SHR: Sub hazard ratio
