# Supplementary material for: Survival analysis and influence of the surgical aggression of a cohort of orthopedic and trauma patients in a non-controlled spread COVID-19 scenario
Source: BMC Musculoskelet Disord. 2021 Jun 28;22:594. doi: 10.1186/s12891-021-04303-8 (PMC8236737; doi:10.1186/s12891-021-04303-8)
Supplement: Supplementary file 1 — Additional file 1. Interview guide. Document use by the researches to guide the telephone interview. [file 12891_2021_4303_MOESM1_ESM.docx]

# Additional file 1: Interview guide

1. Explanation of the study and verbal consent for data collection.
2. Confirmation of type of surgery and date of surgery.
3. Complications related with the surgery.
4. Did the patient attend to any emergency department, consult his/her primary care physician or has been admitted in any hospital after the surgery?
5. Did the patient have any of the following symptoms?
   1. Major symptoms:
      1. Fever.
      2. Dry Cough.
      3. Shortness of Breath.
      4. Anosmia/dysgeusia.
   2. Minor symptoms:
      1. General malaise.
      2. Diarrhea.
      3. Myalgia.
      4. Headache
6. Did the patient need any different treatment apart from the one prescribed by his/her surgeon?
