# Supplementary material for: Survival analysis and influence of the surgical aggression of a cohort of orthopedic and trauma patients in a non-controlled spread COVID-19 scenario
Source: BMC Musculoskelet Disord. 2021 Jun 28;22:594. doi: 10.1186/s12891-021-04303-8 (PMC8236737; doi:10.1186/s12891-021-04303-8)
Supplement: Supplementary file 2 — Additional file 2. Multivariate analysis. The complete STATA data are shown for the following: A Selection of variables. Univariate Cox regression analysis. B Analysis of the confounders and interactions. C Confound assessment. [file 12891_2021_4303_MOESM2_ESM.docx]

# Additional file 2: Multivariate analysis

## Selection of variables. Univariate Cox regression analysis

## Analysis of the confounders and interactions

## *MMax (Maximum model with confounder and interaction variables)

## *MMaxNoInteract (Maximum model without interaction variables)

## *Assessemnt of the significance of interaction variables (likehood ratio test) (p<0.05)

The interactions variables can be removed from the model because the interactions were not significant. (p=0.2778)

*Reference model to be assessed for confounder variables : stcox c.Age i.SurgSev c.HBP c.DM c.CardiolHist c.RenalHist c.RespHi

**C.- Counfound assessement.** Best models with a change in effect (HR=ExpB) less than 10% (Change) compared with the reference model with all the confounder variables and ordered by better precision (lower Range) in the 95% CI.

***Selected Models.** From those models with a change in effect less tah 10% compared with the reference model . it has been selected those models with a narrower 95%CI (lower range) and more parsimonious.

**A) Models with lower change in effect but wider 95%CI and lesser parsimoniuos models**

## B) Adjusted models. Higuer Change in effect, but always less than 10%. More precision (narrowed 95%CI) and more parsomoniuos models.

## C) Most Parsimonious models with higher changes in effect and good precision

## *Fine-Gray Competing risk models
