# Supplementary material for: Survival analysis and influence of the surgical aggression of a cohort of orthopedic and trauma patients in a non-controlled spread COVID-19 scenario
Source: BMC Musculoskelet Disord. 2021 Jun 28;22:594. doi: 10.1186/s12891-021-04303-8 (PMC8236737; doi:10.1186/s12891-021-04303-8)
Supplement: Supplementary file 3 — Additional file 3. Relative risks of COVID-19 diagnosis by Group of Surgery. The complete STATA data are shown for the following: A COVID diagnosis (suspected + probable + confirmed cases). B COVID diagnosis (only probable + confirmed cases). [file 12891_2021_4303_MOESM3_ESM.docx]

# Additional file 3. Relative risks of COVID-19 diagnosis of COVID -19 diagnosis by group of surgery (complete data)

## COVID diagnosis ( suspected + probable + confirmed cases) with correction for zero event

## COVID diagnosis (only probable + confirmed cases) with correction for zero event
