# Supplementary material for: Survival analysis and influence of the surgical aggression of a cohort of orthopedic and trauma patients in a non-controlled spread COVID-19 scenario
Source: BMC Musculoskelet Disord. 2021 Jun 28;22:594. doi: 10.1186/s12891-021-04303-8 (PMC8236737; doi:10.1186/s12891-021-04303-8)
Supplement: Supplementary file 4 — Additional file 4. Distribution of cases in the entire cohort. Table showing the distribution of the evolution of cases in the entire cohort (non-infected; alive; COVID-19 deaths; non-COVID-19 deaths; severe disease). [file 12891_2021_4303_MOESM4_ESM.docx]

# Additional file 4. Distribution of cases in the entire cohort

#
