# Supplementary material for: Survival analysis and influence of the surgical aggression of a cohort of orthopedic and trauma patients in a non-controlled spread COVID-19 scenario
Source: BMC Musculoskelet Disord. 2021 Jun 28;22:594. doi: 10.1186/s12891-021-04303-8 (PMC8236737; doi:10.1186/s12891-021-04303-8)
Supplement: Supplementary file 5 — Additional file 5. Cumulative survival probabilities of the entire cohort according to the K-M method and the actuarial method. The complete STATA data are shown for the following: A Survival list. Entire cohort. B Actuarial method. [file 12891_2021_4303_MOESM5_ESM.docx]

# Additional file 5: Cumulative survival probabilities of the entire cohort with the K-M method and with the actuarial method (complete data)

## Survival list. Entire Cohort.

## Actuarial Method
