# Supplementary material for: Survival analysis and influence of the surgical aggression of a cohort of orthopedic and trauma patients in a non-controlled spread COVID-19 scenario
Source: BMC Musculoskelet Disord. 2021 Jun 28;22:594. doi: 10.1186/s12891-021-04303-8 (PMC8236737; doi:10.1186/s12891-021-04303-8)
Supplement: Supplementary file 6 — Additional file 6. Failure function (cumulative incidence of mortality) in the entire cohort according to the K-M method and the competing risk. The complete STATA data are shown for the following: A Survival function by Group of Surgery. B K-M failure function. Entire cohort. C Failure function. Competing risk. D K-M failure function by Group of Surgery. E Comparison of life tables of survival (actuarial method) by Group of Surgery. [file 12891_2021_4303_MOESM6_ESM.docx]

# Additional file 6: Failure function (cumulative incidence of mortality) in the entire cohort by the K-M method and by the competing risk (complete data)

## Survival function by group of surgery

## K-M Failure function. Entire Cohort.

## Failure function. Competing Risk

##

##


## K-M failure function by Group of Surgery

## Comparison of life tables of survival (Actuarial method) by group of surgery
