# Supplementary material for: Survival analysis and influence of the surgical aggression of a cohort of orthopedic and trauma patients in a non-controlled spread COVID-19 scenario
Source: BMC Musculoskelet Disord. 2021 Jun 28;22:594. doi: 10.1186/s12891-021-04303-8 (PMC8236737; doi:10.1186/s12891-021-04303-8)
Supplement: Supplementary file 7 — Additional file 7. Differences in survival curves by Group of Surgery. The complete STATA data are shown for the following: Log rank, Breslow, and Tare tests for comparing survivor curves by Group of Surgery. [file 12891_2021_4303_MOESM7_ESM.docx]

# Additional file 7: Differences in survival curves by Group of Surgery (complete data)

## Log rank, Breslow and Tare test for comparing survivor curves by group of surgery

##
