# Supplementary material for: Survival analysis and influence of the surgical aggression of a cohort of orthopedic and trauma patients in a non-controlled spread COVID-19 scenario
Source: BMC Musculoskelet Disord. 2021 Jun 28;22:594. doi: 10.1186/s12891-021-04303-8 (PMC8236737; doi:10.1186/s12891-021-04303-8)
Supplement: Supplementary file 8 — Additional file 8. Model assessment. The complete STATA data are shown for the following: A Schonefeld residuals. B Test based on the interaction between survival-time and the independent variables. C Log linear assumption. D Quality of the follow-up. [file 12891_2021_4303_MOESM8_ESM.docx]

# Additional file 8: Model assessment.

***Model diagnosis Porportional HR assumption**

- **A) Schonefeld residuals**

Not significant p value. It is accepted the proportional hazard assumption.

## B)Test based on the interaction between survival-time and the independent variables

## C) Log lineal assumption

stcox c.Age i.SurgSev i.HBP i.RespHist, tvc(Age)

failure _d: EndStatMort == 1

analysis time _t: Tsm_Mortal

Not significant p value (0.406) with the Wald test in the interaction of the quadratic main predictor (_hatsq) for the Age variable with time. Consequently, the it is accepted the log lineal assumption

- **D) Quality of the follow up**

Summary for variables: Tsm_Mortal by categories of: EndStatMort
